# Effects of Long-term Conservation Tillage on Soil Nutrients in Sloping Fields in Regions Characterized by Water and Wind Erosion

**DOI:** 10.1038/srep17592

**Published:** 2015-12-01

**Authors:** Chunjian Tan, Xue Cao, Shuai Yuan, Weiyu Wang, Yongzhong Feng, Bo Qiao

**Affiliations:** 1 College of Agronomy, Northwest A&F University, Yangling, 712100 Shaanxi, China

## Abstract

Conservation tillage is commonly used in regions affected by water and wind erosion. To understand the effects of conservation tillage on soil nutrients and yield, a long-term experiment was set up in a region affected by water and wind erosion on the Loess Plateau. The treatments used were traditional tillage (CK), no tillage (NT), straw mulching (SM), plastic-film mulching (PM), ridging and plastic-film mulching (RPM) and intercropping (In). Our results demonstrate that the available nutrients in soils subjected to non-traditional tillage treatments decreased during the first several years and then remained stable over the last several years of the experiment. The soil organic matter and total nitrogen content increased gradually over 6 years in all treatments except CK. The nutrient content of soils subjected to conservative tillage methods, such as NT and SM, were significantly higher than those in soils under the CK treatment. Straw mulching and film mulching effectively reduced an observed decrease in soybean yield. Over the final 6 years of the experiment, soybean yields followed the trend RPM > PM > SM > NT > CK > In. This trend has implications for controlling soil erosion and preventing non-point source pollution in sloping fields by sacrificing some food production.

The Loess Plateau is the main agricultural region in China. However, because of long-term overuse of resources, the surface soil structure is loose, causing serious soil erosion and restricting sustainable agricultural development in arid regions of the Loess Plateau. Water and soil loss from sloping fields is the main cause of soil erosion and deterioration of the environment on the Loess Plateau. The vast majority of arid regions still use traditional farming techniques, resulting in environmental deterioration, soil and water loss and serious economic costs^1^. Therefore, it is important to use reasonable cultivation measures. In recent years, conservation tillage has been frequently used to reduce water and wind erosion, increase water storage and alleviate ecological pressure^2^,^3^,^4^,^5^,^6^. Many studies of conservation tillage^7^,^8^,^9^,^10^,^11^,^12^,^13^,^14^ have focused on the impact of different tillage methods on soil physical and chemical properties, water conservation, crop yields and economic benefits. However, little research has examined sloping farmland, and especially the effects of long-term conservation tillage on soil nutrients and yield.

The Liu Daogou watershed is located in a region of the Loess Plateau crisscrossed by water and wind erosion in the northern part of Shaanxi Province. The environment in this area is extremely fragile. Not reasonable treatment can disrupt the ecological balance, causing soil erosion or even irreversible desertification^15^,^16^. In this study, we systematically analyzed six consecutive years of data from a long-term experiment conducted from 2008 to 2013 that investigated the effects of different tillage methods on soil nutrients and yield mechanisms. We also explored the most suitable measures for cultivating sloped land areas, providing a reference for managing soil erosion on sloping cultivated land and preventing non-point source pollution in the Loess Plateau area.

## Results

### Changes in soil organic matter content for different tillage methods

**Figure 1:**
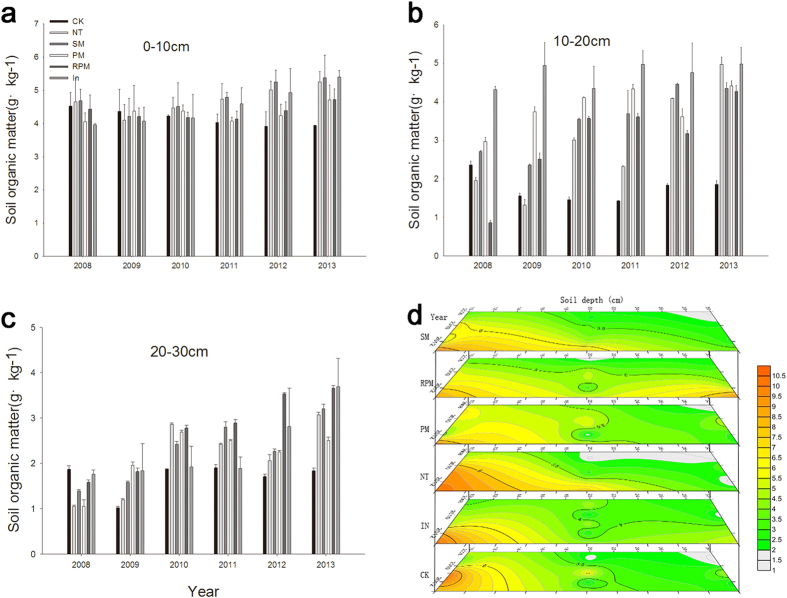
Soil organic matter as affected by tillage treatments, 0–30 cm. (**a**) Soil organic matter over 0–10 cm depth. (**b**) Soil organic matter over 10–20 cm depth. (**c**) Soil organic matter over 20–30 cm depth. (**d**) Dynamic changes of soil organic matter on six treatments at 0–30 cm soil depth. The colorimetric scale colors represent the soil organic matter content (g·kg^−1^).

The organic matter content in soils treated with different tillage methods did not decrease after six consecutive years without fertilization; instead, it increased slowly (Fig. 1a–c). Figure 1d shows the colorimetric scale colors that represent the soil organic matter content. A greater the number of the equivalent lines led to a greater change in organic matter. From 2008 to 2013, the soil organic matter content in the 0–10 cm soil layer increased to varying degrees under different tillage methods except in the CK treatment. However, in the 10–30 cm soil layer, the soil organic matter content for different tillage methods increased and was higher than in the CK treatment. Among them, SM and In have the highest soil organic matter content.

### Changes in the soil total nitrogen content for different tillage methods

**Figure 2:**
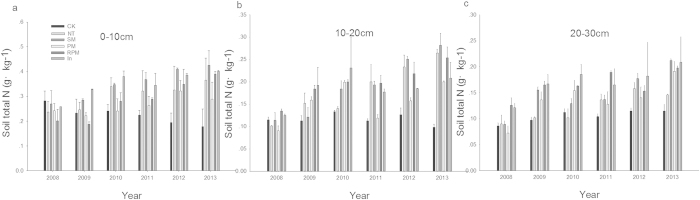
Soil total N as affected by tillage treatments, 0–30 cm (**a**) Soil total N over 0–10 cm depth. (**b**) Soil total N over 10–20 cm depth. (**c**) Soil total N over 20–30 cm depth.

There is a close relationship between total nitrogen and organic matter because 80%–97% of nitrogen is contained in organic matter in surface soils^17^. Soil organic matter and total nitrogen were highly correlated in this study with a correlation coefficient of 0.7885. Figure 2 shows the changes in soil nitrogen content in the 0–30 cm soil layer during the six-year period for each treatment. The total soil nitrogen content increased slowly throughout the entire experiment, and the total nitrogen content in the CK treatment was always lower than in the other treatments. As soil depth increased, the total nitrogen content gradually decreased in all treatments. The total soil total nitrogen content decreased significantly in the 0–10 cm soil layer of the CK treatment. The total nitrogen content was highest in the 0–10 cm soil layer of the In treatment during the first three years, while the SM treatment had the highest total nitrogen content during the last three years. In the 10–20 cm soil layer, the total nitrogen content of the NT treatment increased and remained high; the RPM treatment had the highest total nitrogen content during the first three years, after which this value decreased and stabilized. In contrast to the slow decline in soil organic matter, the total nitrogen content in the surface soils of the CK treatment remained at a low level and then showed a downward trend.

### Changes in soil available nutrient content for different tillage methods

**Figure 3:**
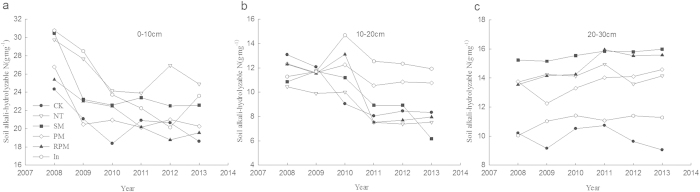
Soil alkali-hydrolyzable N affected by tillage treatments, 0–30 cm (**a**) Soil alkali-hydrolyzable N over 0–10 cm depth. (**b**) Soil alkali-hydrolyzable N over 10–20 cm depth. (**c**) Soil alkali-hydrolyzable N over 20–30 cm depth.

Changes in soil available nitrogen content Fig. 3 shows that the soil available nitrogen content associated with different tillage methods decreased quickly in the 0–10 cm soil layer and more slowly in the 10–20 cm soil layer. However, in the 20–30 cm soil layer, the soil available nitrogen content increased slowly with each farming year. The soil available nitrogen content was highest from the shallow to the deep soil layers of the NT, RPM and SM treatments, showing that these treatments effectively improved or preserved the available nitrogen in the soil.

### Changes in soil availhable phosphorus content

**Figure 4:**
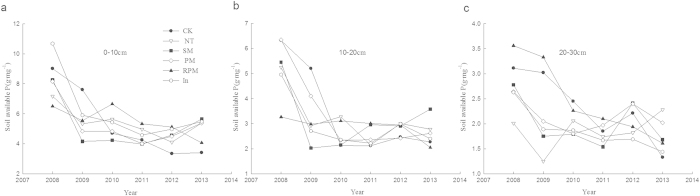
Soil available P affected by tillage treatments, 0–30 cm (**a**) Soil available P over 0–10 cm depth. (**b**) Soil available P over 10–20 cm depth. (**c**) Soil available P over 20–30 cm depth.

In contrast to soil available nitrogen, Fig. 4 shows that the available phosphorus content decreased rapidly each year and remained at a very low level. The soil available phosphorus content gradually decreased in the RPM treatment, but in the SM treatment, the available phosphorus content decreased rapidly in the beginning and then increased slowly. In the 0–10 cm soil layer, the soil available phosphorus content was lower in all treatments compared to the CK treatment during the first two years. After two years, the available phosphorus content decreased rapidly in the CK treatment, but stabilized and increased in the other treatments with the exception of the RPM treatment. The changes in soil available phosphorus content in the 10–20 cm soil layer are similar to those in the 0–10 cm soil layer. The variation trend of 10–20 cm and 20–30 cm was similar to that of 0–10 cm.

### Changes in available potassium content

**Figure 5:**
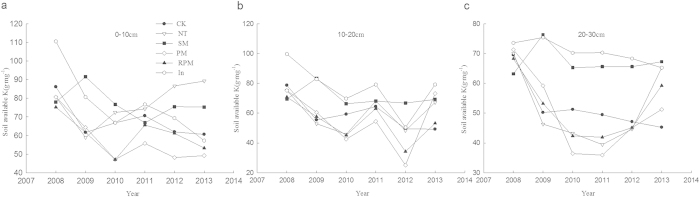
Soil available K affected by tillage treatments, 0–30 cm (**a**) Soil available K over 0–10 cm depth. (**b**) Soil available K over 10–20 cm depth. (**c**) Soil available K over 20–30 cm depth.

Soil available potassium content decreased over the first two years except in the SM treatment, where it increased slightly and then gradually became stable after the third year. In the 0–10 cm soil layer, soil available potassium content in the NT treatment always increased and was the highest (Fig. 5a). In the 10–30 cm soil layer, the SM and RPM treatments were the highest (Fig. 5b,c). The soil available potassium content in the SM treatment decreased slowly and remained relatively high, possibly because supplementary available potassium was associated with the SM treatment. The lowest soil available potassium content occurred in the PM treatment in most years. For all tillage methods, the available potassium content decreased gradually with increasing soil depth.

### Changes in crop production

**Figure 6:**
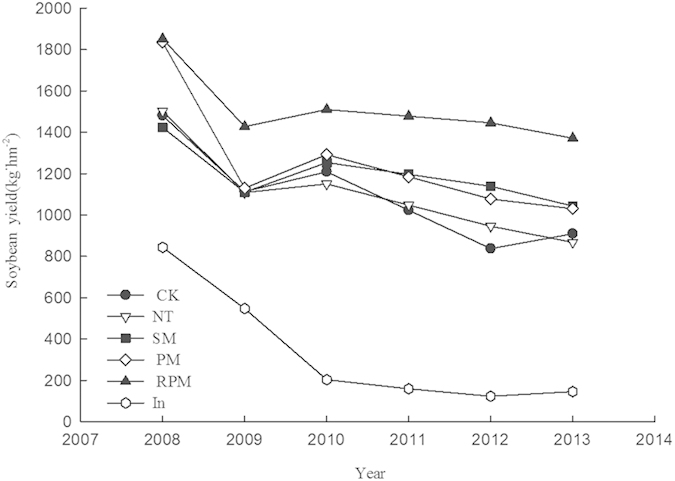
Yield of soybean as affected by tillage treatments, 2008–2013.

Figure 6 shows the changes in crop production due to different tillage methods. Soybean production decreased drastically during the first two years and then gradually stabilized at a low level. In most years, soybean production in the NT treatment was higher than in the CK treatment. Soybean production in the In treatment was lower than in all other treatments, including the CK treatment, but soybean production in CK treatment was lower than in the remaining treatments. The crop yields for the SM, PM, and RPM treatments increased by 14.79%, 13.35%, and 50.84% in 2013 compared to the CK treatment. Soybean production in the RPM treatment was the highest throughout the experiment and was significantly different from the other treatments. Soybean production in the six tillage methods can be ranked in the order RPM > PM > NT > CK > SM > In for 2008; in 2013, the order was RPM > SM > PM > CK > NT > In. This change in relative production among the treatments suggests that the RPM and SM treatments resulted in an increase in soybean yields after six years.

## Discussion

As demonstrated by Yang Peipei *et al.*^18^, the soil organic matter and the total N, P and K content of soils in conservation tillage show the phenomenon of surface accumulation. This occurs because the tillage layer is not destroyed in no-tillage methods; similarly, covering the surface layer with straw leads to nutrient enrichment in the plough layer. SM and In increase the surface soil nutrient, possibly because the degradation of straw and *Medicago sativa* litter can add surface organic matter and increase the soil organic matter content. The NT treatment reduced the frequency of soil disturbance and caused less damage to the soil structure, which reduced the loss of soil organic matter during erosion. Therefore, the topsoil organic matter content of NT was higher. The surface soil organic matter content in the PM and RPM treatments was relatively low compared to the other treatments in the long term, which may be because the coverage is not conducive to crop leaves and other residues returned to the soil, thereby inhibiting soil biological activity. In addition, the soil organic matter content of each treatment decreased with depth. Our results show that organic matter, total nitrogen and available nutrients decreased with depth in soils treated with conservation tillage, which is consistent with the above results.

Studies have demonstrated that the organic carbon and nitrogen content of soil decreases prior to stabilizing as a result of long periods without fertilization^19^. Our test results over six years showed that without the input of artificial fertilizers, the organic matter and nitrogen content of soil subjected to conventional tillage treatments decreased, which is consistent with the above results. Conversely, the organic matter and nitrogen content of conservation tillage treatments did not decline with increasing cultivation age; rather, they increase gradually, which supports the above results. This result may be related to the effects of crop straw. Straw is rich in high-carbon materials such as cellulose and lignin, which are major sources of soil organic matter. Straw decomposition releases carbon dioxide, which can promote soil microbial immobilization or release and mineralize inorganic nitrogen, ultimately forming soil organic matter^20^. Soybeans, which are nitrogen fixers, were grown for several years in the experimental fields, and may have provided soil nitrogen.

Research has demonstrated that tillage disturbance changes the conditions of decomposition, causing the soil respiration rate to increase and the organic matter content to decrease^21^. However, our results do not reflect these changes, possibly because conservation tillage alleviated this disturbance to some extent. Zou Congming *et al.*^22^ suggested that straw mulching was conducive to the accumulation of soil alkali-hydrolysable nitrogen, but the ridge tillage method does not affect it. Our study shows obvious changes in the soil available nitrogen content in soils subjected to different tillage methods. The NT, RPM and SM treatments improved the available soil nitrogen content and preserved the nutrients in the soil. Our observation of annual decreases in the surface soil available nitrogen content differs from the results of previous studies, most likely because soil erosion in sloping land eliminates many available nutrients. However, the soil available nitrogen content gradually increased in the deeper soil layers, possibly because of the addition of nitrogen from soybean nitrogen fixation. According a study by Li Shiqing^23^, long-term mulching deteriorates soil ecological conditions, causing excessive mineralization of soil organic matter and lessening soil water-stable aggregates and total microbial quantity. This phenomenon is not clearly demonstrated in our study because of the limited study duration.

A study by T. Quine^24^ showed that alkali-hydrolysable nitrogen, soil available potassium and soil organic matter all decrease because of erosion. Our results showed that alkali-hydrolysable nitrogen, available phosphorus and effective potassium decreased more slowly under conservation compared to conventional tillage. Therefore, we suggest that conservation tillage methods can protect against soil erosion. The total organic matter and nitrogen did not decrease with the number of farming years but rather gradually increased. This may be due to the supplementation of organic matter from straw and litter and the addition of nitrogen from fixation by legumes.

Tillage methods may have effects on soil structure, soil water retention and fertilizer maintenance. However, seasonal crop production can be attributed to the soil environment. J. Gómez *et al.*^25^ found that compared to conventional tillage, crop yields were higher and crop quality was better under no-tillage mulching. Our results demonstrate that soybean yields were higher in other treatments compared with conventional tillage, except for alfalfa-soybean intercropping. The highest soybean yields were observed under ridging film mulching, while the lowest soybean yields were observed under alfalfa-soybean intercropping. Ridging film mulching can change the microtopography of the field as well as increasing the surface cover. These factors can help conserve soil water and fertilizer. Ridging film mulching creates a good soil environment for crop growth, improving water use efficiency over the growth cycle and promoting plant growth conditions and the accumulation of photosynthetic production. Because alfalfa consumes a larger amount of water under alfalfa-soybean intercropping, interplanting crops is disadvantageous in terms of water and fertilizer, and results in lower yields. No tillage production is also a poor choice and is only slightly different from conventional tillage. P. De Vita *et al.*^26^ showed that no-tillage methods did not increase crop yields.

## Conclusions

Our long-term experiment demonstrated that in a region subject to water and wind erosion on the Loess Plateau, conservation tillage can increase soil nutrients when no fertilizers were used. Compared with traditional tillage treatments, conservation treatments led to a gradual decrease and then stabilization and increase in soil organic matter, total nitrogen, alkali-hydrolyzable nitrogen, available phosphorus, and available potassium. Straw mulching treatments helped to preserve heat as well as to supplement the nutrients in the tillage layer. Plastic-film mulching improved the soil environment, was more conducive to fertilizer and water retention, and increased crop yields. Therefore, we suggest that conservation tillage methods have broad prospects in the basin; they can help reduce soil erosion, improve soil fertility, protect the environment and control non-point source pollution.

## Materials and Methods

### Details of the experimental field

**Figure 7:**
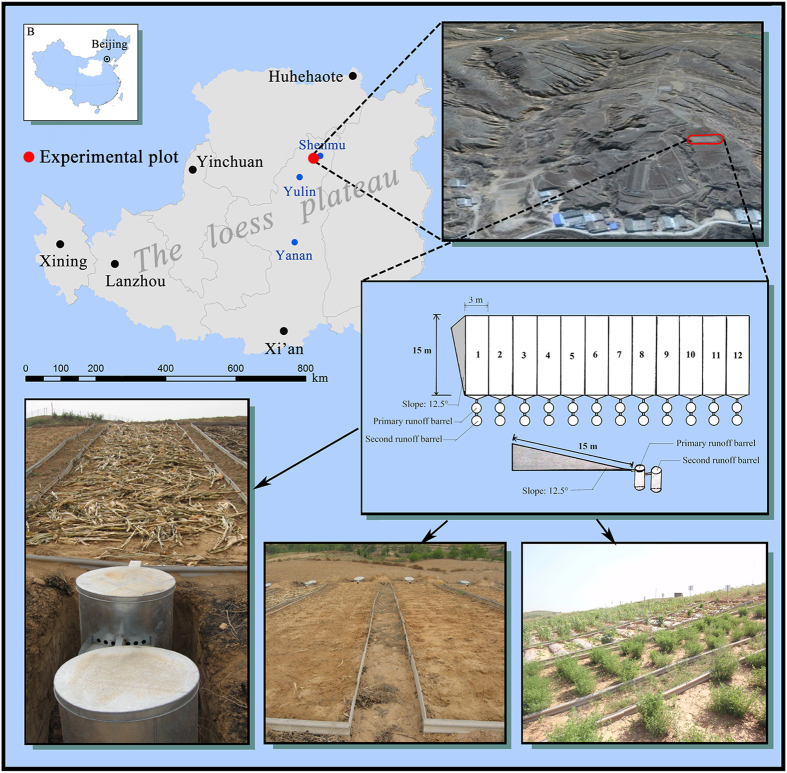
The location of reseach in Shenmu erosion and environmental testing station of Institute of Soil and Water Conservation (110°21′–110°23′E, 38°46′–48°51′N). The test field was established in May, 2008, each runoff field with length of 15 m, width of 3 m, the slope of 12.5 degrees. Photographs were taken by Chunjian Tan. The map was plotted by Arcgis 9.3 (URL:http://www.esri.com).

The experiment was conducted from 2008 to 2013 on farmland sloped at 12.5 degrees located at the Shenmu erosion and environmental testing station of the Institute of Soil and Water Conservation (110°21′–110°23′E, 38°46′–48°51′N). The station is in the Liu Daogou watershed of western Shen Mu county, Yu Lin city, Shaanxi province, and is approximately 14 km north of the Great Wall in the transition zone of the Loess Plateau between the Mu Us Desert and forest steppe to typical arid steppe (Fig. 7). Wind and water erosion crisscross this region. The basin has a semi-dry climate and is located in a warm temperate zone with an average annual evaporation capacity of 785.4 mm, and an annual precipitation of 437.4 mm. Over 6–9 months, rainfall accounted for 77.4% of the total precipitation, the annual average temperature was 8.4 °C and the frost-free period was 169 days. The experimental field was planted with potatoes for the local farmers; Table 1 lists the soil nutrient values that were determined in 2008.

### The experimental design

The Jindou 21 and Zhong Mu No. 1 fields were used for the experiment. Soybeans were sown in mid-April. The seeding rate of soybean was 45 kg ha^−1^. The alfalfa as green manure was widely used in the experimental region and was not harvested during the experiment. The test field was built in May of 2008; each runoff field had a length of 15 m, a width of 3 m, and a slope of 12.5 degrees. The total area of the experimental field was 540 m^2^. The experiment was laid out in a randomized block design. Six treatments (traditional tillage, no-tillage farming, straw mulching, plastic mulching, ridge mulching, and intercropping) with two replications each were established for a total of 12 experimental plots. The experiment was conducted with no fertilizer added (Table 2).

### Sample collection, analysis and data processing

Each year, following the soybean harvest at the end of September, five sampling points were randomly selected from each plot. Soil samples were collected from depths of 10 cm, 20 cm, and 30 cm and were subsequently blended and dried in the laboratory. Each sample was filtered through 1 mm and 0.25 mm screens into plastic bags for soil nutrient analysis. Quarterly after maturity, representative areas from each treatment were selected to measure crop yields. Plow layer soil (0–30 cm) was obtained for soil nutrient measurements using conventional methods^27^. Organic matter was extracted by the K_2_CrO_7_-H_2_SO_4_ heat treatment, and residue was determined using the FeSO_4_ titration of potassium dichromate method. Nitrogen was determined through Kjeldahl nitrogen fixing, and available nitrogen was determined by the alkaline diffusion method. Available phosphorus was determined by the NaHCO_3_ extraction colorimetric method and the molybdenum blue method, and potassium determination was performed by the flame photometric method.

## Additional Information

**How to cite this article**: Tan, C. *et al.* Effects of Long-term Conservation Tillage on Soil Nutrients in Sloping Fields in Regions Characterized by Water and Wind Erosion. *Sci. Rep.* **5**, 17592; doi: 10.1038/srep17592 (2015).

## Figures and Tables

**Table 1:** Basic chemical properties of soil in 2008.

Organic matte (g·kg^−1^)	Total N (g·kg^−1^)	Total P (g·kg^−1^)	Total K (g·kg^−1^)	Alkali-hydrolysable N (g·mg^−1^)	Available P (g·mg^−1^)	Available K(g·mg^−1^)
6.44	0.50	0.44	6.70	10.56	3.10	62.90

**Table 2:** Treatments description.

Treatments	Description	Code
Conventional tillage	Traditional tillage.	CK
No-tillage	No tillage,no coverage with any debris.	NT
Straw mulching	Traditional tillage,straw mulching. Straw coverage with 5500 kg/hm^2^.	SM
Plastic mulching	Ploughing and laying plastic mulch.Turns the soil surrounding the plastic, then planting between the plastic.	PM
Ridging and Plastic mulching	Planting on both sides of ridge, then mulching on the entire ridge, no coverage on furrow; space between neighboring ridge top midlines 100 cm, ridge height 30 cm, planting crops on both sides of ridge.	RPM
Interplanting	Intercropping between alfalfa plants.	In
